# Parents’ Willingness to Vaccinate Children against COVID-19 in Saudi Arabia: A Cross-Sectional Study

**DOI:** 10.3390/vaccines10020156

**Published:** 2022-01-21

**Authors:** Soukaina Ennaceur, Mohammed Al-Mohaithef

**Affiliations:** 1 Department of Public Health, College of Health Sciences, Saudi Electronic University, Jeddah 11673, Saudi Arabia; 2 Department of Public Health, College of Health Sciences, Saudi Electronic University, Riyadh 13323, Saudi Arabia; m.almohaithef@seu.edu.sa

**Keywords:** COVID-19, vaccination, pandemic, children, parents’ willingness, Saudi Arabia

## Abstract

Objectives: The present study aimed to investigate parents’ willingness to vaccinate their children under the age of 18 with a COVID-19 vaccine. Methods: This cross-sectional study was conducted in Saudi Arabia from January 2021 to March 2021. The univariate analysis using Mann–Whitney *U*-test, *t*-test, and chi-squared/Fisher’s exact test was performed to identify sociodemographic factors associated with the acceptance of COVID-19 vaccine in children. Factors with statistical significance (*p* < 0.05) were analyzed using multivariate regression analysis to determine the variables affecting parents’ decisions to vaccinate children. Results: Overall, 44% (167) of parents reported that they would accept vaccinating their children with a COVID-19 vaccine. Young (86; 22.7%), married (135; 35.6%), and Saudi (114; 30%) parents seemed to be more concerned about their children being infected. Parents who intended to vaccinate themselves (OR: 0.599, 95% CI: 0.367–0.980) and who trust the healthcare system (OR: 0.527, 95% CI: 0.327–0.848) reported greater acceptance of children’s vaccination. Among parents, the most frequent (40.9%) reason for vaccinating children was to prevent infection in other family members. What may underlie this result is that some parents understand that children can carry pathogens from persons in school to thoseat home. The most frequent (22.2%) reason for refusing vaccination was concerns about the side effects of the vaccine. Conclusions: Parents have differing opinions on frequencies and risks of coronavirus disease transmission and medical complications and of effectiveness and adverse effects of a vaccine. These results could be of use in designing public health information campaigns and health promotion programs based on perceived parental behavior and positive attitudes.

## 1. Introduction

The World Health Organization (WHO) has reported more than 185 million confirmed cases of COVID-19 worldwide, including approximately 4 million deaths as of 7 July 2021 [1]. An effective strategy to mitigate the morbidity and mortality of COVID-19 and to ensure higher levels of immunity in the population involves the development of an effective and safe vaccine for all populations, including children. Since the start of the COVID-19 pandemic, a high number of vaccines have been developed [2]. The WHO declared on 11 July 2021 that 107 and 184 vaccine candidates were in clinical and pre-clinical stages, respectively [3], and at least 13 vaccines have been approved and administrated through four platforms [4]. As of 7 July 2021, approximately 3 billion vaccine doses have been administered worldwide [1]. In Saudi Arabia, the Saudi General Food and Drug Authority (FDA) issued the first emergency use authorization for the Pfizer-BioNTech vaccine on 10 December 2020 [5]. On 18 February 2021, the FDA approved the use of the AstraZeneca COVID-19 vaccine [6]. The Ministry of Health established a well-organized vaccine program to provide the COVID-19 vaccine to all citizens and residents in the country. A COVID-19 free vaccine booking service was provided to obtainan appointment in advance to receive the vaccine, at no cost, at the nearest vaccine center.

However, an effective and safe vaccine is not sufficient to stop the pandemic. A high level of vaccine acceptance among the general population is also a mandatory factor. It has been found that during pandemics, effective vaccine campaigns rely on several factors such as (i) the personal risk perception of the harm or the adverse effects of the vaccine [7,8], (ii) the positive recommendation of the vaccine by a healthcare professional [9], and (iii) the knowledge of the disease and the vaccine development process [8,10,11]. Moreover, some studies demonstrated a significant association between sociodemographic characteristics of the participants and the vaccine perception during a pandemic. For example, age and gender were found to be associated with the acceptance of a pandemic vaccine [9,10]. There was no clear trend regarding other demographic factors such as education, occupation, social status, and marital status [12]. Other studies found that participants who showed acceptance of previous vaccination (national vaccination such as the influenza vaccine) have a greater intention to be vaccinated with a pandemic vaccine [7,8,9,13,14,15]. In a previous study, conducted during the second wave of the COVID-19 pandemic in Saudi Arabia, our research group found that only 53.3% of the participants had shown interest in being vaccinated if an effective vaccine became available [16]. Since the government introduced new legislation on 1 August 2021 that established vaccination as a prerequisite to gain entrance to all public places, activities, events, private and governmental facilities, and the use of public transportation across the country [17], a downward trend was observed in the curve of COVID-19 cases, which tended toward a stable state and an increasing demand of the vaccine [18]. Moreover, on 23 June 2021, the Ministry of Health announced that more than 16.8 million COVID-19 vaccine doses had been administrated through 587 vaccination centers, covering 70% of the adult population in Saudi Arabia [18]. To expand the vaccine administration, a new phase of the National COVID-19 Vaccine Campaign was implemented in June 2021 to administer the Pfizer vaccine to the 12-to-18 age group [18]. This vaccination phase aimed to help children return to their normal lives and attend schools.

Many factors such as fear about vaccine safety and risks of children becoming infected by the virus affected parents’ acceptance of the new COVID-19 vaccination program. Early studies on the COVID-19 pandemic showed that few numbers of children were infected by the virus [19].

To the best of our knowledge, no investigations have studied parents’ willingness to vaccinate their children against COVID-19 in Saudi Arabia. The present study aimed to identify factors associated with parental acceptance and refusal to enroll their children aged between 12 and 18 years in a COVID-19 vaccination program.

## 2. Materials

### 2.1. Study Designand Participants

This cross-sectional study was conducted in Saudi Arabia between January 2021 and March 2021. The ethical approval of the study was obtained from the Ethics Committee of the Saudi Electronic University (SEUREC-CHS20118). A questionnaire was used to determine the prevalence of parents’ acceptability of the COVID-19 vaccine for their children and to study the reasons behind their opinions and views. The questionnaire was developed as part of a larger study assessing parents’ perception and acceptability of a COVID-19 vaccine for themselves and their children during the COVID-19 pandemic. Inclusion criteria were voluntary participation, parents of a child under the age of 18, and living in Saudi Arabia. Parents were provided with the necessary information about the study—namely, the objectives of the study, the research team identity, their right to discontinue their participation, confidentiality, and data protection, and they were informed that only complete registered data would be considered for data analysis.

Once they agreed to participate in the study, the parents provided their consent in an online questionnaire. The survey validity was tested using a pilot version for all items completed by 15 parents from the target population (colleagues from the SEU) who provided feedback regarding the comprehensibility of the questions and the time to complete the survey. Those 15 parents were not part of the actual survey.

According to the inclusion criteria, only 379 parents out of 658 participants (participation rate = 57.6%) were eligible to be included in the present study. We developed an open-access online survey using the SurveyMonkey program, which is a widely used online survey platform in Saudi Arabia. For security purposes, a mass message was sent along with the questionnaire link to the respondents via social media: Twitter, Facebook, WhatsApp, and email lists. A request to share the survey hyperlink was also sent to the participants. The survey tool was available both in Arabic and English.

### 2.2. Data Collection

The self-administered questionnaire consisted of 23 questions that took approximately 10 min to answer and included sociodemographic characteristics of the parents (age, gender, number of family members, socioeconomic status, education level, etc.), previous seasonal influenza vaccination of the parents, and whether they refused, postponed or had a vaccine for a child or themselves despite doubts about its efficacy. The questionnaire also enquired about the parents’ perception and willingness to vaccinate their children under the age of 18 with a COVID-19 vaccine. The responses were “Yes”, “No” and “Not sure”. The parents were also asked about their willingness to sign up, or any member of their family, for a COVID-19 vaccine if available. Responses to this question were“Yes”, “No” and “Not sure”. If the answer was “Yes”, five reasons were offered to the parents: “I am worried about getting sick”; “There are many COVID-19 cases in my society”; ”I am a member of a target group recommended to receive the vaccine, such as a healthcare worker, adult aged over 50, or a pregnant woman”; “My healthcare provider recommended to take the COVID-19 vaccine”;”I want to keep others in my household safe from getting sick”. In case the parent answered “No”, 14 reasons were offered: “The vaccine is too new”; “Its effectiveness is not known”; “I am worried about its side-effects”; “I avoid most vaccines”; “I do not think I will be infected with the COVID-19 virus”; “I do not think that the COVID-19 virus will cause serious illness even if I am infected”; “I do not think the vaccine will prevent infection”; “It is inconvenient to take a vaccine that requires multiple doses”; “I am not a member of a target group to receive the vaccine”; “My religion prevents vaccination”; “It might cost too much”; “I do not know where to get vaccinated”; “I have immunity because I was already infected with COVID-19”; “I do not have health insurance”. If the answer was “Not sure”, four reasons were provided: “The vaccine is too new”; “Its effectiveness is not known”; “Its side effects are not known”; “Its doses are not known”. Open-text boxes were provided for the three answers to help parents give more reasons on vaccine acceptance for themselves or a member of their family.

### 2.3. Statistical Analysis

The statistical analysis of the data was performed using the IBM, SPSS V. 28.0 package program(IBM Corporation, Armonk, NY, USA). A *p*-value less than 0.05 was considered for statistical significance. Categorical variables on sociodemographic characteristics of the parents were expressed using descriptive statistics and frequencies. A univariate analysis using the Mann–Whitney *U*-test (for non-normal continuous variables), *t*-test (for normal distribution of continuous variables), and chi-squared test (for categorical variables) was performed to determine parental characteristics associated with their willingness to vaccinate children under the age of 18 with a COVID-19 vaccine. Then, multivariate logistic regression analysis was conducted for the variables with significance (*p* < 0.05) in the univariate analysis to evaluate the odds ratios of the acceptability of a COVID-19 vaccine for children.

## 3. Results

In total, 379 parents responded to the survey. The mean age of the participants was 31.9 (SD ± 8.2) years. Approximately, equal numbers of males (191–50.4%) and females (188–49.6%) were present in the study. The majority of parents were Saudi citizens (83.6%), married (60.7%), with large families with more than four members (79.6%), with a good socioeconomic status (82.6%), and from the northern, eastern, and central regions of Saudi Arabia (70.1%) (Table 1). In addition, most of the respondents were graduates (46.7%), compared with the other categories (15.6% had a level of education equal to high school or below, 21.4% of participants were post-graduates, and 16.3% had a diploma). Only 16.4% of those who completed the survey are working in the healthcare system.

Among 379 parents, 167 (44%) were willing to allow their children to receive a COVID-19 vaccine, but 212 (56%) were not willing or were hesitant to vaccinate their children.

The univariate analysis showed that greater acceptance of vaccination of children with a COVID-19 vaccine was associated with parents’ age (*p* = 0.017), nationality (*p* = 0.025), marital status (*p* = 0.005), number of family members (*p* = 0.042), profession (*p* < 0.001), and education level (*p* < 0.001). No statistically significant association was registered between the parents’ acceptance of the COVID-19 vaccine administration to their children under the age of 18 and the parent’sgender (*p* = 0.617), the socioeconomic status of the family (*p* = 0.492), the place of residence (*p* = 0.144), and whether the parent is working or not in a healthcare system (*p* = 0.208).

Comparison between sub-groups showed that a higher level of parental acceptability of children being vaccinated with a COVID-19 vaccine was found among parents aged between 31 and 40 years (22%), compared with old parents (6.6%); among Saudi citizens (35.6%) when compared with foreign residents in the county (8.7%); among married parents (30%), compared with parents living separately, divorced, or widowed; among parents having four or more members in their families (33.2%) when compared withthose living in small families (1.3%); among workers (30.4%) when compared with unemployed parents (13.7%); among graduated and post-graduated parents (30.5%), compared with parents with high school education or below (6.3%) (Table 2).

Table 3 shows the parents’ attitudes and opinions associated with their willingness to vaccinate their children under the age of 18. The results demonstrated that parents who reported a higher level of acceptance to vaccinate themselves (72.3%) were more willing to give a COVID-19 vaccine to their children than parents who were not willing to accept being vaccinated (6.8%) (*p* < 0.001). Moreover, parents who were willing to participate in a COVID-19 clinical trial (78.3%) were more willing to vaccinate their children with a COVID-19 vaccine than those refusing participation in such a trial (21.7%) (*p* < 0.001). Parents who highly trusted the healthcare system (69.6%) were more likely to vaccinate their children with a COVID-19 vaccine than those with low trust (51.1%) (*p* = 0.018).

The parents were asked about their preference for the COVID-19 vaccine brand (domestic or imported). A total of 77.8% of the parents were more likely to prefer an imported vaccine. Parents who reported this preference had a higher intention of protecting their children through vaccination (50.0%) than those preferring a domestic COVID-19 vaccine (42.7%) (*p* = 0.003). When questioned about their concern of somebody in their families or themselves becoming infected with the coronavirus, 21.9% of the parents indicated such a concern. Those who did have that concern were more willing for a COVID-19 vaccine to be administrated to their children (62.7%) than those without any concerns (39.5%) (*p* = 0.002). To understand the parental vaccination routine under the national vaccination program, parents were asked if they (i) had refused a vaccine for themselves or a child because they considered the vaccination useless or dangerous; (ii) had postponed a vaccine for themselves or a child even if the vaccine had been recommended by a physician; (iii) had administrated a vaccine for themselves or a child despite having doubts about the vaccine’s efficacy. Parents who declared that they refused a vaccine for themselves or a child (82.2%) were more likely to refuse vaccination of their children with a COVID-19 vaccine than those who accepted their children to receive a vaccine under the national vaccination program (51.1%) (*p* = 0.001). The results also showed that 44.3% of parents had received the seasonal flu vaccines. Parents who had received the seasonal flu vaccine responded that vaccinating their children with a COVID-19 vaccine was the most protective strategy (33.3%), compared with those who were not enrolled in a seasonal flu vaccination program (16.6%) (*p* = 0.013).

Factors with a *p*-value < 0.05 in the univariate analysis were included in a multivariate logistic analysis to identify the predictors impacting parents’ acceptability of a COVID-19 vaccine to be administrated to their children under the age of 18. The multivariate analysis (Table 4) showed that parental willingness to vaccinate themselves (OR = 0.599, 95% CI 0.367–0.980, *p* = 0.041), parents’ trust in the healthcare system (OR = 0.527, 95% CI 0.327–0.848, *p* = 0.008), parental concerns about someone in their families or themselves becoming infected with the coronavirus (OR = 0.397, 95% CI 0.228–0.693, *p* = 0.001), and parental attitude to refuse a vaccine for themselves or a child because they consider vaccines useless or dangerous (OR= 4.067, 95% CI 1.872–8.833, *p* = 0.001) are behavioral and attitudinal factors associated with the willingness to vaccinate children with a COVID-19 vaccine. No parental characteristics were found to be associated with their willingness to accept a COVID-19 vaccine to be administrated to their children.

**Figure 1:**
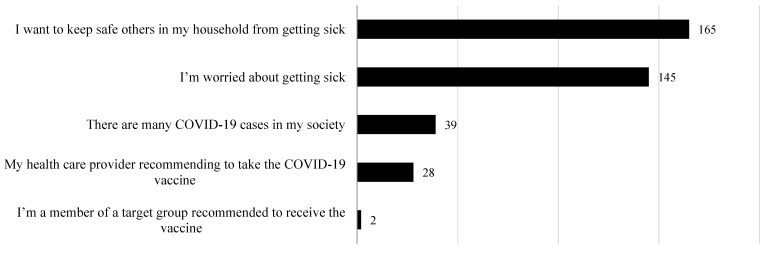
Parental reasons for accepting to vaccinate themselves or one of their family members with a COVID-19 vaccine.

Figure 1 represents the reasons given to the parents regarding COVID-19 vaccine acceptance for themselves and their children. Of those parents who reported their willingness to vaccinate their children with a COVID-19 vaccine, the most important reason was to protect other members of their household from becoming ill (40.9%), followed by concerns about becoming ill themselves (35.3%).

Additionally, 4.1% of parents filled in the opentext box to explain their willingness to vaccinate themselves or their children with the COVID-19 vaccine. Table 5 shows the parents’ reasons. Some parents cited that vaccination is the best way to protect themselves and their families against the infection by COVID-19 and to limit the spread of the disease within the community. Others considered that if the health authority (the Ministry of Health) recommended a COVID-19 vaccine, they would accept to be vaccinated along with their children. One woman was worried about her husband who suffers from a chronic disease. She reported her great willingness to vaccinate her husband and her family members to protect him.

**Figure 2:**
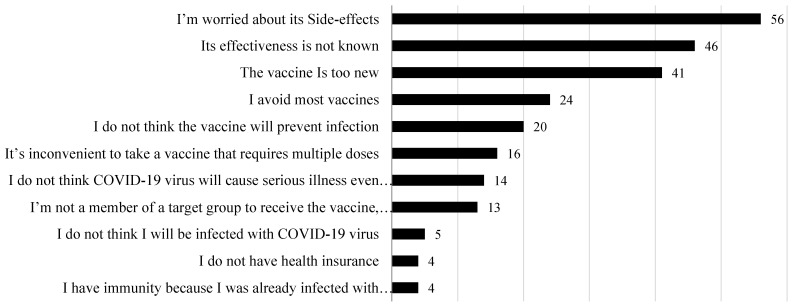
Parental reasons for not accepting to vaccinate themselves or one of their family members with a COVID-19 vaccine.

Parents who reported their refusal to vaccinate themselves and their children were also given reasons to refuse a COVID-19 vaccine if it becomes available (Figure 2). The highest concern about a COVID-19 vaccine was the side effects (22.9%) of the vaccine and its effectiveness (18.7%), followed by concerns about the novel aspect of the vaccine (16.7%). Other parents explained their refusal to participate in a vaccine campaign was due to their rejection of all national vaccination (9.7%).

Moreover, an open-text response was given to the parents who intended to reject a COVID-19 vaccine for themselves and their children. The collected answers are presented in Table 6. Some parents reported their doubts about the reliability of the information related to the COVID-19 vaccine’s effects on human fertility. Others expressed their concerns about the COVID-19 vaccine because they suffer from allergies. Other parents simply reported that they were worried about being vaccinated.

## 4. Discussion

To limit the spread of the COVID-19 pandemic, a safe and effective vaccine needed to be developed and administrated to all the population, including children. During the first period of the COVID-19 vaccine approvals, none were recommended for use on children. Recently, some vaccine sponsors have suggested the enrollment of children as young as 12 years and above in COVID-19 vaccine campaigns [20]. The family’s role is to understand the benefits of a COVID-19 vaccine as an effective response to the pandemic.

The present study is one of the first to be conducted in Saudi Arabia to understand the parents’ willingness and attitudes toward enrolling their children under the age of 18 in a COVID-19 vaccination campaign and to provide vaccination program planners with preliminary information for future COVID-19 vaccination plans for children.

Parental attitudes toward a COVID-19 vaccine for their children seem to be positive, as shown in the present study, given that 44% of parents reported that they will accept to vaccinate their children if an effective COVID-19 vaccine is available. However, in our sample, the willingness to vaccinate children was lower than rates registered in recent studies conducted in the United States (63%) [21], China (72.6%) [22], and England (48.2%) [23]. In addition, an international study including six nations found that 65% of parents intend to vaccinate their children against COVID-19 [24]. In our previous study, the percentage of parents who were willing to vaccinate themselves was 53% [16], which could explain the present findings.

The main factors found to be associated with parents’ willingness to vaccinate their children with a COVID-19 vaccine were the parents’ ageyoung parents, rather than older parents, were more likely to accept a COVID-19 vaccine to be administered to their children. Saudi parents seemed to be more concerned about their children being infected with the virus and consequently showed a higher intent to vaccinate their children. Parents living together showed more motivation to protect their children through a COVID-19 vaccine. Families with a high number of members had the intention to vaccinate their children against OVID-19. Those who were employed and those with higher education levels showed greater willingness to accept children’s vaccinations. Similar factors were found to be associated with parental willingness to vaccinate their children with a COVID-19 vaccine in previous studies [22,24,25]. Vaccination is the appropriate strategy to be applied in the country to help people return to their normal life (work, travel, celebrate holidays, visit restaurants, etc.), and more importantly, enables children to return to school and their normal everyday life. It has been shown that children’s vaccination against COVID-19 could also reduce rare severe COVID-19 pediatric cases and multisystem inflammatory syndrome in children [26]. Consequently, parents are encouraged to accept their children to be vaccinated with a COVID-19 vaccine since this protects children against the disease and will reduce transmission in the home, family stress levels, and economic difficulties. Moreover, to achievewidespread children immunization, effective health promotion programs should be established, a positive attitude toward the COVID-19 vaccine must be promoted, health communication messages must be addressed to parents to raise their awareness of the importance of increasing community immunization through vaccinations, and confidence in vaccine supply should be enhanced. Previous studies reported several factors impacting parents’ agreement to vaccinate their children with a COVID-19 vaccine. These factors include children’s protection [23,24], protection of others from the disease [23], high levels of fear and anxiety due to the pandemic [27,28,29], and the fact that vaccination is an effective strategy to control COVID-19 spread [28,30,31]. In the present study, parents’ willingness to vaccinate their children was impacted by similar factors. Parents who intended to vaccinate themselves or to participate in a COVID-19 clinical trial showed a positive attitude and considered children’s vaccination. Trust in the healthcare system in Saudi Arabia was found to be another facilitator towards children vaccination against COVID-19. Interestingly, parents’ behavior regarding the vaccination, in general, did not change with the spread of COVID-19. Parents who refused to vaccinate their children under the national vaccination program still refused to vaccinate their children against COVID-19. The results of the present study show that approving the participation of children in a COVID-19 vaccine campaign is associated with the vaccine brand (domestic or imported). Parents reported great acceptance of children’s vaccination if an imported vaccine becomes available.

Studies investigating parental rejection or hesitancy of a COVID-19 vaccine showed several determinants such as“asymptomatic course of COVID-19 in children” [16,24,25] and “uncertainties about the effectiveness and safety of the COVID-19 vaccine” [23,32,33]. In the present study, the main reasons to refuse a COVID-19 vaccine to be administered to the children were fear about side effects and vaccine effectiveness. Concerns about vaccine effectiveness and safety were related to the rapid development process of the vaccine and exposure to negative information about the vaccine through social media. In fact, during the COVID-19 pandemic, a large spread of misinformation and inaccurate data were spread through the media [34], causing a decrease in parents’ confidence and acceptance of the COVID-19 vaccine. This situation could be controlled through transparent communication about the development of the vaccine and its safety and effectiveness among populations including children. The identification of this misinformation in a limited time is the responsibility of the public health authorities to protect populations from being impacted by such unverified rumors.

Although this is the first study to be conducted in Saudi Arabia to investigate parents’ willingness and attitudes towards children’s vaccination with the COVID-19 vaccine, some limitations have to be discussed. First, this study was part of a larger investigation conducted in the Saudi general population. The inclusion criteria limited the number of participants since only parents with children under the age of 18 were included, which caused the non-representativeness of the sample in the regions where the survey was administrated. Second, selection bias might be another limitation since only participants using the internet and social media were invited to participate through the online survey. Participants with no internet access were not part of the study. Third, the objectives of the study were to determine factors associated with parents’ willingness to accept children’s vaccinations promptly to help public health authorities and decisionmakers in implementing adequate children vaccination programs. This caused a lack of innovation in the applied methodology. Fourth, the cross-sectional study design did not enable us to investigate causality. Fifth, the use of a self-administered survey and the difficulty to conduct face-to-face interviews (due to social distancing) led to a recall bias since responses to the survey questions could not be verified. Last, data were collected during the second COVID-19 wave (January–March 2021), parents’ willingness might change over time, and the strategies of the health authorities could impact parents’ attitudes toward their vaccination intentions for their children.

In conclusion, the rate of parents’ willing to administer a COVID-19 vaccine to children under the age of 18 was acceptable in the present study. Many strategies could be applied to encourage parents to accept to vaccinate their children and to achieve herd immunity, for example, including the COVID-19 vaccine in the school vaccination program, communicating clear and transparent information about the COVID-19 vaccine development process and the expected side effects of the vaccine, and establishing health promotion programs based on positive parental attitudes and perceived behavioral control.

## Figures and Tables

**Table 1:** Sociodemographic characteristics of the parents assessed for their willingness to vaccinate their children with a COVID-19 vaccine.

Characteristics		Number (%)
Age	18–30	165 (43.5%)
	31–40	164 (43.3%)
	>40	50 (13.2%)
Gender	Female	188 (49.6)
	Male	191 (50.4)
Nationality	Saudi	317 (83.6%)
	Non-Saudi	62 (16.4%)
Marital status	Married	230 (60.7%)
	Unmarried ^a^	149 (39.9%)
Family members	1	6 (1.6%)
	2	22 (5.8%)
	3	49 (13%)
	≥4	302 (79.6%)
Socioeconomic status	Low	36 (9.5%)
	Medium	313 (82.6%)
	High	30 (7.9%)
Occupation	Not working	146 (38.5%)
	Self-employed	16 (4.2%)
	Private sector	70 (18.5%)
	Governmental sector	147 (38.8%)
Education	High school or below	59 (15.6%)
	Diploma	62 (16.3%)
	Graduate	177 (46.7%)
	Post-graduate	81 (21.4%)
City of residence	Northern and eastern regions	114 (30%)
	Southern and western regions	80 (21.1%)
	Central region	152 (40.1%)
	Other ^b^	33 (8.8%)
Working in a healthcare system	No	317 (83.6%)
	Yes	62 (16.4%)

^a^ Unmarried: single, separated, divorced, and widowed. ^b^ Other: didnot specify the region.

**Table 2:** Parental characteristics associated with the willingness to vaccinate their children with a COVID-19 vaccine.

Characteristics	Willing to Vaccinate the Child	Not Willing to Vaccinate the Child ^a^	*p*-Value
	167 (44%)	212 (56%)	
Age			0.017
18–30	57 (15%)	108 (28.5%)	
31–40	86 (22.7%)	78 (20.5%)	
>40	25 (6.6%)	25 (6.6%)	
Gender			0.617
Female	76 (20.0%)	111 (29.2%)	
Male	92 (24.2%)	100 (26.3%)	
Nationality			0.025
Saudi	135 (35.6%)	182 (48.0%)	
Non-Saudi	33 (8.7%)	29 (7.6%)	
Marital status			0.005
Married	114 (30.0%)	117 (30.8%)	
Unmarried	24 (6.3%)	94 (24.8%)	
Family members			0.042
1	5 (1.3%)	1 (0.2%)	
2	9 (2.3%)	13 (3.4%)	
3	28 (7.4%)	21 (5.5%)	
≥4	126 (33.2%)	176 (46.4%)	
Socio-economic status			0.492
Low	15 (3.9%)	21 (5.5%)	
Medium	138 (36.4%)	175 (46.1%)	
High	15 (3.9%)	15 (3.9%)	
Occupation			<0.001
Not working	52 (13.7%)	94 (24.8%)	
Self-employed	4 (1.0%)	11 (2.9%)	
Private	23 (6.0%)	47 (12.4%)	
Government	89 (23.4%)	59 (15.5%)	
Education			<0.001
High school or below	24 (6.3%)	35 (9.2%)	
Diploma	28 (7.3%)	34 (8.9%)	
Graduate	63 (16.6%)	113 (29.8%)	
Post-graduate	53 (13.9%)	29 (7.6%)	
City of residence			0.144
Northern and eastern	47 (12.4%)	67 (17.6%)	
Southern and western	42 (11.0%)	38 (10.0%)	
Central	63 (16.6%)	89 (23.4%)	
Other	16 (4.2%)	17 (4.4%)	
Working in HCS ^b^			0.208
No	32 (8.4%)	30 (7.9%)	
Yes	136 (35.9%)	181 (47.7%)	

^a^ Not willing: total of parents not sure and parents not willing to vaccinate their child/ren with a COVID-19 vaccine. ^b^ HCS: healthcare system.

**Table 3:** Parents’ attitudes and opinions associated with their willingness to vaccinate their children with a COVID-19 vaccine.

Parents’Attitudesand Opinions Associated with a COVID-19 Vaccine Administration to Their Children				
	Willing to Vaccinate the Child	Not Willing to Vaccinate the Child ^a^	Total	*p*-Value
	167 (44%)	212 (56%)	379	
Willingness to vaccinate themselves with a COVID-19 vaccine				
No	11 (6.8%)	151 (93.2%)	162 (42.7%)	<0.001
Yes	157 (72.3%)	60 (27.6%)	217 (52.2%)	
Willingness to participate in a COVID-19 vaccine clinical trial				
No	132 (39.6%)	201 (60.4%)	333 (87.8%)	<0.001
Yes	36 (78.3%)	10 (21.7%)	46 (12.1%)	
Trust in the healthcare system				
No	108 (51.1%)	103 (48.2%)	211 (56.6%)	0.018
Yes	117 (69.6%)	51 (30.4%)	168 (44.3%)	
Confidence in domestic vaccines				
No	78 (35.6%)	141 (64.4%)	219 (57.8%)	0.084
Yes	90 (56.2%)	70 (43.8%)	160 (42.2%)	
Willingness to vaccinate with a domestic or imported brand vaccine ^b^				
No	126 (42.7%)	169 (57.3%)	295 (77.8%)	0.003
Yes	42 (50%)	42 (50%)	84 (22.2%)	
Concerned about being infected or someone in their family with the COVID-19 virus				
No	117 (39.5%)	179 (60.5.5%)	296 (78.1%)	0.002
Yes	52 (62.7%)	31 (37.3%)	83 (21.9%)	
Refused a vaccine for themselves or a child because they considered it useless or dangerous				
No	158 (48.9%)	165 (51.1%)	323 (85.2%)	0.001
Yes	10 (17.8%)	46 (82.2%)	56 (14.8%)	
Postponed a vaccine for themselves or a child, recommended by a physician				
No	152 (45.1%)	185 (54.9%)	337 (88.9%)	0.370
Yes	16 (38.0%)	26 (62.0%)	42 (11.0%)	
Had a vaccine for themselves or a child despite doubts about its efficacy				
No	138 (42.2%)	189 (57.8%)	327 (86.2%)	0.142
Yes	30 (57.7%)	22 (42.3%)	52 (13.8%)	
Received a seasonal flu vaccine				
No	35 (16.6%)	176 (83.4%)	211 (55.7%)	0.013
Yes	56 (33.3%)	112 (66.7%)	168 (44.3%)	

^a^ Not willing: total of parents not sure and parents not willing to vaccinate their child/ren with a COVID-19 vaccine. ^b^ No: parents refusing a domestic vaccine and Yes: parents accepting a domestic vaccine.

**Table 4:** Predictors related to the parents’ acceptance to vaccinate their children with a COVID-19 vaccine.

Variable	Adjusted Odds Ratio (95% CI)	*p*-Value
Age		
18–30	0.779 (0.303–2.003)	0.604
31–40	1.202 (0.583–2.480)	0.618
>40	1	
Nationality		
Saudi	1.539 (0.790–2.997)	0.205
Non-Saudi	1	
Marital status		
Married	1.492 (0.754–2.953)	0.250
Unmarried	1	
Family members		
1	0.941 (0.155–5.705)	0.948
2	0.708 (0.264–1.902)	0.493
3	0.554 (0.266–1.157)	0.116
≥4	1	
Occupation		
Not working	0.720 (0.343–1.514)	0.387
Self-employed	1.188 (0.353–4.000)	0.781
Private	0.734 (0.348–1.551)	0.418
Government	1	
Education		
High school or below	1.180 (0.452–3.081)	0.736
Diploma and Graduate	0.855 (0.407–1.798)	0.680
Post-graduate	1	
Willingness to vaccinate themselves with a COVID-19 vaccine		
No	0.599 (0.367–0.980)	0.041
Yes	1	
Willingness to participate in a COVID-19 vaccine clinical trial		
No	1.235 (0.585–2.607)	0.057
Yes	1	
Trust in the healthcare system		
No	0.527 (0.327–0.848)	0.008
Yes	1	
Willingness to vaccinate with a domestic/imported brand vaccine		
No	1.135 (0.616–2.091)	0.684
Yes	1	
Concerned about someone in their family or themselves being infected with coronavirus		
No	0.397 (0.228–0.693)	0.001
Yes	1	
Refused a vaccine for themselves or a child because it is useless or dangerous		
No	4.067 (1.872–8.833)	<0.001
Yes	1	
Received a seasonal flu vaccine		
No	0.976 (0.551–1.730)	0.935
Yes	1	

**Table 5:** Quotes for willingness to accept a COVID-19 vaccine.

Quotes
“Vaccine is the best protective medication against the spread of the disease in the community”.
“I trust the authorities; if they command the administration of a COVID-19 vaccine, I will participate in the vaccination campaign”.
“The vaccination is an effective strategy to not only protect myself but also to protect my family”.
“It’s my responsibility to protect myself and my family, so I will vaccinate myself if a COVID-19 vaccine becomes available”.
“I trust science and the decision-makers in my country and I know that if my country approves the use of the COVID-19 vaccine, it will be the right decision”.
“My husband is suffering from a chronic disease and I think that being vaccinated will protect him”.
“I was infected by the coronavirus, and I don’t want to experience the same infection for a second time”.
“Because vaccination is mandatory for traveling”.
“To protect myself from infection by any other COVID-19 variant”.

**Table 6:** Quotes from those not willing to participate in a COVID-19 vaccination campaign.

Quotes
“I doubt the existence of a disease name COVID-19”.
“I suffer from allergies and the health authorities recommended not to be vaccinated”.
“I think that vaccination will not enhance the body’s immune system”.
“The clinical trial stages of the COVID-19 vaccine were not sufficient”.
“The virus has unstable genetic material and can change to other more active variants”.
“The mortality rates of exposure to COVID-19 are not exceeding those of normal influenza”.
“I am simply worried about getting vaccinated.”
“I doubt information regarding the effects of the COVID-19 vaccine on human fertility”.
“I do not trust the vaccine producers”.

## Data Availability

All data generated or analyzed during this study are included in this published article.
